# A Technical Review of Minimally Invasive Mitral Valve Replacements

**DOI:** 10.1007/s13239-014-0203-9

**Published:** 2014-11-25

**Authors:** Georgia L. Preston-Maher, Ryo Torii, Gaetano Burriesci

**Affiliations:** Cardiovascular Engineering Laboratory, UCL Mechanical Engineering, Torrington Place, London, WC1E 7JE UK

**Keywords:** Mitral valve, Transcatheter heart valve replacement, TAVI, TMVI

## Abstract

Mitral regurgitation is one of the most common forms of heart valve disorder, which may result in heart failure. Due to the rapid ageing of the population, surgical repair and replacement treatments, which have represented an effective treatment in the past, are now unsuitable for about half of symptomatic patients, who are judged high-risk. Encouraged by the positive experience with transcatheter aortic valves and percutaneous reconstructive mitral treatments, a number of research groups are currently engaged in the development of minimally invasive approaches for the functional replacement of the mitral valve. The first experiences have clearly demonstrated that the approach is feasible and promising, though significant progress is still required in the prostheses design and implantation procedures before the treatment can establish as a safe and effective solution. This review analyses the devices currently at a most advanced stage of development, describing their main features and the technical solutions that they adopt in order to respond to the functional requirements of the most challenging of the heart valves.

## Introduction

Mitral regurgitation (MR) is one of the most common forms of heart valve disorder, occurring when blood leaks from the left ventricle into the left atrium. This results from the failure of apposition of the mitral valve leaflets, due to degenerative or functional causes. Degenerative MR is associated with alterations of the mitral valve leaflet structure, whereas functional MR occurs due to changes in shape of the heart chambers or damage to the heart muscle (e.g., from myocardial infarction) resulting in annular dilatation and papillary muscle displacement, which compromises correct leaflet coaptation.

If untreated, MR results in increased afterload and heart failure risk. It is estimated that symptomatic MR affects over four million Europeans and a similar number of Americans^29^,^36^,^42^ and, due to the continuous ageing of the western population, the number of cases is expected to rise significantly in the future. In fact, MR prevalence increases with age, rising from 0.5% for young patients (18–44-year-old) to 9.3% for patients older than 75 years.^42^,^52^ Therefore, a significant proportion of symptomatic patients are in their late age, with relevant co-morbidities and previous surgery.^20^,^29^,^33^,^47^ As a result, up to 50% of them are currently declined for surgery because they are judged too ill or weak to withstand the stress of the invasive treatment.^29^,^40^,^50^

In recent years, the growing need for less invasive therapeutic approaches has led to the development of a number of reconstructive percutaneous treatments for MR. However, these procedures mainly contribute to alleviate the symptoms and are only suitable for very specific forms of mitral valve disease and anatomic subsets. The possibility to perform a complete percutaneous mitral valve replacement still represents an unmet need, which would provide enormous advantages for a vast and enlarging patients’ population.^15^,^30^

Transcatheter valve replacement has already been successfully applied to the treatment of pulmonary and aortic valves.^22^ However, despite the relevance of the described clinical need, the application of this approach to the mitral valve has been attempted only recently, and is still in its infancy. This delay is justified by the more severe technical challenges associated with the geometry of the anatomical site and the more critical loading conditions.

This review presents a technical description of the most advanced solutions for percutaneous functional replacement of the mitral valve that are currently reported in the literature. In particular, the alternative approaches adopted to meet the functional requirements are analyzed and discussed, identifying the main areas that still require improvement in order to transform this disruptive technology into a sustainable treatment.

## Percutaneous Mitral Valve Repair

The first percutaneous mitral valve procedure can be dated back to the early 1980s, when Inoue and colleagues performed the first balloon valvuloplasty for treatment of mitral stenosis,^28^ accessing the valve region *via* antegrade venous route (with transseptal puncture). This approach has quickly become the solution of choice for congenital stenotic mitral valves^8^,^39^ in patients with isolated mitral stenosis and suitable anatomy.^53^ However, this disease is now uncommon in developed countries and the procedure is not free from complications, including failure to relieve stenosis, formation of MR and systemic embolization.^2^,^53^ Mitral valve regurgitation represents a much more common disease in the western world,^14^ which affects patients often untreatable surgically.^29^,^40^,^50^ Hence, a number of minimally invasive procedures have recently been developed to target this disease. These are essentially reconstructive treatments (valve *repair*) that aim to improve the leaflets apposition by remodeling one of the functional substructures. Below, they are classified based on the substructure that they target to achieve their function.

### Leaflets Plication Procedures

The main percutaneous approach currently adopted for mitral repair is based on the edge-to-edge technique. This consists in the apposition of the central portion of the anterior and posterior mitral valve leaflets to create a double-orifice valve with reduced leaflet excursion and reduced regurgitation.^1^ This is achieved by manipulating a grasping clip (MitraClip, Abbott Vascular)^12^ or suturing device (Mobius, Edwards).^10^,^15^ The main limitation of this procedure is it being restricted to patients without severe dilatation of mitral annulus, relatively normal leaflets, mitral valve prolapse and central regurgitation.^6^ Moreover, clinical results indicate the inability of the technique to eliminate regurgitation in all patients.^21^ In fact, the edge-to-edge technique was developed as an adjunct to standard surgical repair procedures, and its use as a stand-alone technique is still debated.^6^,^13^

In 2008 the Abbott’s MitraClip percutaneous repair device obtained CE marking, followed by FDA approval in 2013. This remains the only percutaneous reconstructive device currently available on the market.

### Annulus Reshaping Procedures

Other popular approaches aim to restore the leaflets coaptation by reshaping the mitral annulus. In the first solutions, this was achieved by implanting into the coronary sinus a stent-like device (such as the Carillon, Cardiac Dimensions and the Monarc, Edwards) that forces the reshaping of the posterior region of the annulus, producing an approximation of the mitral valve leaflets.^32^,^35^ However, this approach is considerably limited by the great anatomical variability of the coronary sinus, and is not applicable in about half of the patients. Lastly, the consequence of long-term placement of such an aggressive prosthesis into the coronary sinus, whose walls are very thin, is still unknown and raises some concern.^2^,^6^,^12^

Other devices achieve the reduction of the anteroposterior diameter by applying an epicardial pressure that modifies the shape of the left ventricle and, consequently, the annulus. This is obtained by tethering cords passed into the ventricle (iCoapsys, Myocor) or inflating silicone bands placed around the atrioventricular groove (BACE, Mardil).

Alternative recent methodologies replicate more closely surgical annuloplasty, which achieve leaflet coaptation by undersizing the annulus perimeter. These are based on transannular or subannular cinching by means of sutures that are anchored and tethered (e.g., Mitralign System, Mitralign and Accucinch System, Guided Delivery Systems), or by anchored Dacron bands of adjustable length (Cardioband, Valtech). The main limitation of these approaches is that they only allow partial cinching (posterior leaflet only).^10^,^26^

Other solutions achieve the annulus undersizing by shrinking its collagen fibers with the heat generated by radio frequencies or ultrasound (e.g., QuantumCor Device, QuantumCor and ReCor Device, ReCor Medical). The drawback of this approach is the potential over-constriction or undercorrection, as well as possible damage to the surrounding structures.^10^ The long-term outcome for these devices is still unknown, and their development has been discontinued.

## Minimally Invasive Heart Valve Functional Replacements

As described above, percutaneous repair treatments generally only relieve the symptoms of very specific forms of mitral valve disease and anatomic subset, and are still surpassed in efficacy by surgical repair.^25^,^38^ A minimally invasive mitral valve functional replacement would allow the treatment of a broader spectrum of patients, disease etiologies and anatomical variations, with significant benefits for the substantial patient population unable to undergo invasive surgery.^15^,^30^ This approach would reduce both the procedural and recovery time for heart valve replacements,^41^ with significant potential for cost saving^43^ as well as more accessible procedure both in terms of patient populations, pathologies and global location,^11^ surpassing the relatively excessive risk factors and resource-intensive requirements of conventional open heart surgical replacements. Moreover, as shown in the case of the pulmonary valve, they can represent an excellent bridge treatment before surgery for children.

### Pulmonary and Aortic Valve Experience

The first human valve replacement using a percutaneous procedure was reported in 2000 by Professor Philipp Bonhoeffer,^9^ who successfully implanted a valved stent in the pulmonary artery prosthetic conduit of a 12-year-old boy with stenosis and insufficiency. The valve was an 18 mm bovine jugular vein valve, sutured to a NuMed CP platinum-iridium stent. The prosthesis received CE Mark approval in 2006 and FDA approval under Humanitarian Device Exemption in 2010, and is now widely used.

The approach was soon adopted for the treatment of the aortic valve, with the first human implantation described by Dr Alain Cribier and colleagues in 2002. They delivered a bovine pericardial valve sewn into a stainless steel stent using a venous trans-septal (antegrade) approach. The patient, who had been judged to be too weak to withstand the stress of open-heart surgery, was a 57-year-old man who had presented in cardiogenic shock due to severe calcific aortic stenosis with a bicuspid aortic valve. Since then, transcatheter aortic valve implantation (TAVI) has established as the treatment of preference for calcified aortic valves in high risk patients.^22^,^24^,^54^ Two valve devices, the Edwards SAPIEN (a direct evolution of the prosthesis implanted by Dr Cribier) and the Medtronic CoreValve, have been in the European market since 2007 (FDA approval was granted in 2011 and 2014, respectively) and a number of second generation devices are already emerging.

### Adaptation of Transcatheter Aortic Valve Implantation Technology for the Mitral Position

Despite the pertinent clinical need,^6^,^7^ the translation of transcatheter valve technologies to the mitral position has been hindered by the need of alternative engineering strategies, taking into account the complex morphology of the valve, the higher transvalvular pressure and the larger size of the required devices.^48^ Also, this application necessitates the adaptation of the delivery and deployment methods, sizing algorithms, intraprocedural imaging, failure modes and post procedural assessment parameters, all of which are unique to the mitral position.

### Main Devices Under Development and Structural Components

A number of research groups and companies are currently working on the adaptation of percutaneous valve solutions specifically for the mitral position. In this section, the transcatheter mitral valve implants (TMVI) known at a most advanced stage of development are described and discussed. These include:

- *CardiAQ Prosthesis* (CardiAQ Valve Technologies Inc., Winchester, Massachusetts, US)
- *Cardiovalve* (Valtech Cardio Ltd, Or Yehuda, Israel)
- *Double-Crowned Mitral Valve Implantation* (Zhejiang University, Hangzhou, China & Centre Hospitalier Universitaire Vaudois (CHUV), Lausanne, Switzerland)
- *Endovalve* (Micro Interventional Devices, Langhorne, Pennsylvania, US)
- *FORTIS* (Edwards Lifesciences, California, US)
- *Gorman* (The Trustees of The University of Pennsylvania, Philadelphia, US)
- *HighLife* (HighLife Medical Inc., California, US)
- *Medtronic Transcatheter Mitral Valve* (Medtronic Inc., Minneapolis, US)
- *MitralSeal* (Avalon Medical Ltd., Stillwater, Minneapolis, US)
- *MitrAssist* (MitrAssist Medical Ltd, Misgav, Israel)
- *MiVAR* (Trinity College Dublin, EIRE)
- *NaviGate Cardiac Structures* (NaviGate Cardiac Structures Inc., Cleveland, Ohio, US)
- *Tendyne* (Tendyne Medical Inc., Baltimore, Maryland, US)
- *Tiara* (NeoVasc Inc., Richmond, British Columbia, Canada)

A schematic illustration of the devices and a description of their main features are reported in Table 1.

**Table 1:**
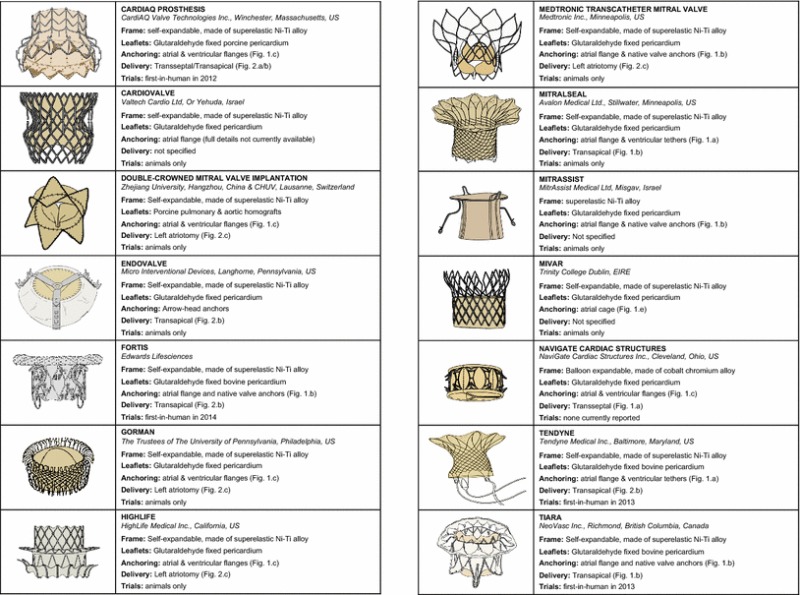
Representation of the devices known at a most advanced stage of development and description of their main features

As in all prosthetic heart valves, in order to restore unidirectional blood flow through the cardiac chambers, the devices are comprised of an occluding component, a supporting structure and a securing means that avoids valve dehiscence. The presence of a sealing component to minimize paravalvular leakage is also preferable, especially for mitral valve applications.

The occluding component of all described solutions consist of three membrane leaflets (apart from the *MitrAssist* which has two asymmetrical leaflets) made from glutaraldehyde fixed bovine or porcine pericardium, sewn onto a supporting frame. This functional solution is optimal for percutaneous valves because the flexibility of the membranes allows them to be folded easily. Moreover, membrane valves can operate in different configurations, accommodating the specific anatomical shapes and dimensions. This feature offers the possibility to adapt the valve to an implantation site whose dimensions and tissue elasticity cannot be accurately determined and are likely to change during the working life of the device.

All of the supporting frames, with the exception of the *NaviGate Cardiac Structures*, are *self*-*expanding* structures made of nitinol. This is a near-equiatomic Ni–Ti alloy that exhibits enhanced recoverable elastic strains up to 8% (about 20 times larger than for stainless steel), commonly referred to as super-elastic behavior. Thanks to this property, the valves are delivered after crimping them inside a covering sheath, which is then pulled back once the prostheses have reached the anatomical site. This allows the frames to re-expand towards their unstressed configurations. The self-expanding approach provides the ability to better adapt to geometrical changes that may occur in the implantation site during the valve life.

In the case of the *NaviGate Cardiac Structures* the frame is made from a *balloon*-*expandable* Co–Cr alloy. During the implantation, the valve is collapsed around an empty balloon with a crimping device, which plastically deforms the frame by producing plastic hinges. The valve is then re-deformed to the expanded configuration by inflating the balloon with a liquid solution. Balloon-expandable approaches allow the operator to administer the deployment pressure, but in the case of the mitral valve they require particular care to avoid excessive deformation of the aortic valve. Moreover, they are typically less suitable for the implementation of retrievable procedures.

#### Anchoring Approach

The anchoring methods required for percutaneous mitral valves are distinct from those used in TAVI devices, due to the more irregular and dynamic morphology. In fact, the substantial amount of calcium present in a stenotic native aortic valve can generate the large radial reaction forces that are commonly used to fix aortic prostheses. In the case of the mitral valve, the stiffening mineral is normally insufficient to provide radial forces able to secure the device. Moreover, high levels of radial forces would not be advisable, as they could cause left ventricular outflow obstruction (LVOTO)^18^ due to the native anterior leaflet being pushed radially into the LVOT, as well as possible aortic valve impairment, due to the device extending into the aorto-mitral curtain.^51^

**Figure 1:**
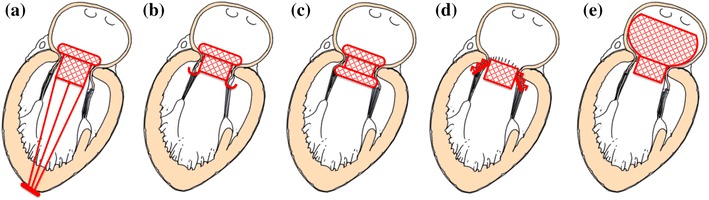
Percutaneous mitral valve anchoring systems. (a) Atrial flange and ventricular tethers, (b) atrial flange and native valve anchors, (c) atrial and ventricular flanges, (d) subannular hooks, and (e) atrial cage

Hence, anchoring in percutaneous mitral valves is commonly achieved by application of counteracting axial forces, tensioning the device between a proximal and a distal constraints. In the case of the *Tendyne* and *MitralSeal* the proximal constraint is represented by a flange, which lies flat against the atrial surface of the native mitral annulus, while the valve is fixed distally to the apex of the left ventricle, through a set of tensioned threads (Fig. 1a). Although these threads look similar to *chordae tendineae*, they have the function to anchor the frame, with no direct action on the valve leaflets. This solution may contribute to the reduction of paravalvular leakage (PVL), thanks to the presence of the atrial flange pressed on the inlet wall. A potential drawback might be represented by possible variations in the tension applied to the distal threads in the event of left ventricle remodelling.

An alternative approach is adopted in the *FORTIS*, *Gorman*, *Medtronic*, *MitrAssist*, and *Tiara*, which instead of threads use ventricular distal anchors that grasp the free margins of the native leaflets (Fig. 1b). The *Fortis* is secured by two ventricular tabs that capture the aortic and mural leaflets, combined with an atrial flange. The *Gorman* valve is secured using upper and lower wire-weave flanges. The *Tiara* uses ventricular tabs that capture the aortic and mural leaflets, combined with three atrial flanges that anchor the device on the right and left fibrous trigones and native posterior leaflet. An additional feature of the *Tiara* anchoring system is its saddle-shape, designed to better conform the native annulus.^31^ However, this makes the implantation of the device more challenging, as the saddle shaped device demands accurate orientation. A potential advantage of clamping the native leaflets could derive by reducing the risk of LVOTO, though the long-term effect of the force acting on the subvalvular components still needs to be ascertained.^45^

The *Double*-*Crowned Mitral Valve* and the *HighLife* adopt clamping mechanisms that engage with the upstream and downstream sides of the mitral annulus, reducing the interaction with the subvalvular apparatus (Fig. 1c). This is achieved by squeezing the mitral annulus between two Z-stents. The deployment of the *Double*-*Crowned Mitral Valve Implantation* requires the presence of an annuloplasty ring in the mitral annulus, thus making its application limited to only a sub-set of patients. The *HighLife* relies on the alignment of a groove with the annulus to anchor the device and provide a seal between the left atrium and the ventricle. The same securing principle is used by the *CardiAQ Prosthesis*, *Cardiovalve* and *NaviGate Cardiac Structures*, which incorporate two sets of barbs or ‘wings’ protruding from the main body to improve the grasp. The advantage of this approach is a reduced interference with the subvalvular apparatus, which avoids potential issues associated with re-modeling. However, the solution does not prevent LVOTO, and the presence of barbs may represent a risk for surrounding structures such as the coronary sinus.

In the case of the *Endovalve*, fixation of the device is provided by a series of anchors around the edge of the valve, consisting of a solid core and flexible barb (Fig. 1d). The flexible barb hugs the core, thereby enabling the anchors to pierce the mitral valve tissue as the device is deployed. Once inserted inside the tissue, the barb springs away from the core, forming an arrowhead which prevents retraction and ensures that the anchors do not disengage. This anchoring method could be effective in preventing PVL, but it does not facilitate multiple deployment or retrieval of the device. Therefore, incorrect release of the device would foreseeably require open-heart surgery.

An alternative approach is provided by the *MiVAR* valve, which relies on an atrial fixation system (Fig. 1e); based on a nitinol cage that conforms to the atrial chamber, preventing axial displacement of the valve. A potential disadvantage of this design is that, each time the left ventricle contracts, the associated apical-basal motion^30^ and pressure gradient may cause the device to move relative to the wall of the left atrium, the consequences of which are to be determined. This solution makes the valve operation totally independent from any remodelling that may occur to the subvalvular apparatus or left ventricle, and could help to reduce PVL. Though, the presence of the anchoring cage could impair the atrial function, as well as the aortic valve.

#### Prevention of Regurgitation

Contrary to standard surgical valves, which are normally sutured onto the aortic annulus (after dissection of the native leaflets), transcatheter valves are expanded into the diseased valve leaflets. This may result in the presence of gaps between the prosthesis and the surrounding native tissues, with consequent PVL.

PVL has been identified as a major shortcoming of TAVI, and its impact is potentially more prominent in the mitral position due to the higher transvalvular pressure difference.^17^ Therefore it is of paramount importance that any leakage around the edge of the device is actively mitigated with specific design solutions. Most of the devices, including the *Cardiovalve*, *Endovalve*, *FORTIS*, *HighLife*, *Medtronic*, *Mitraseal*, *Tendyne* and *Tiara*, prevent PVL by using a fabric flange sutured onto the aortic portion of the metal frame. A unique method is employed by the *Gorman* valve, which relies on the flexibility of a nitinol wire-weave stent body to conform to the complex host geometry and create a seal. The *CardiAQ Prosthesis*, *Double*-*Crowned Mitral Valve*, *MitrAssist*, *NaviGate* and *MiVAR* do not report any specific measure taken to mitigate PVL.

#### Delivery

TMVI need to conform to the irregular anatomy of the site and larger orifices than TAVI. This requires more material for the valve components, which translates into larger diameters of the collapsed device and wider access routes.

**Figure 2:**
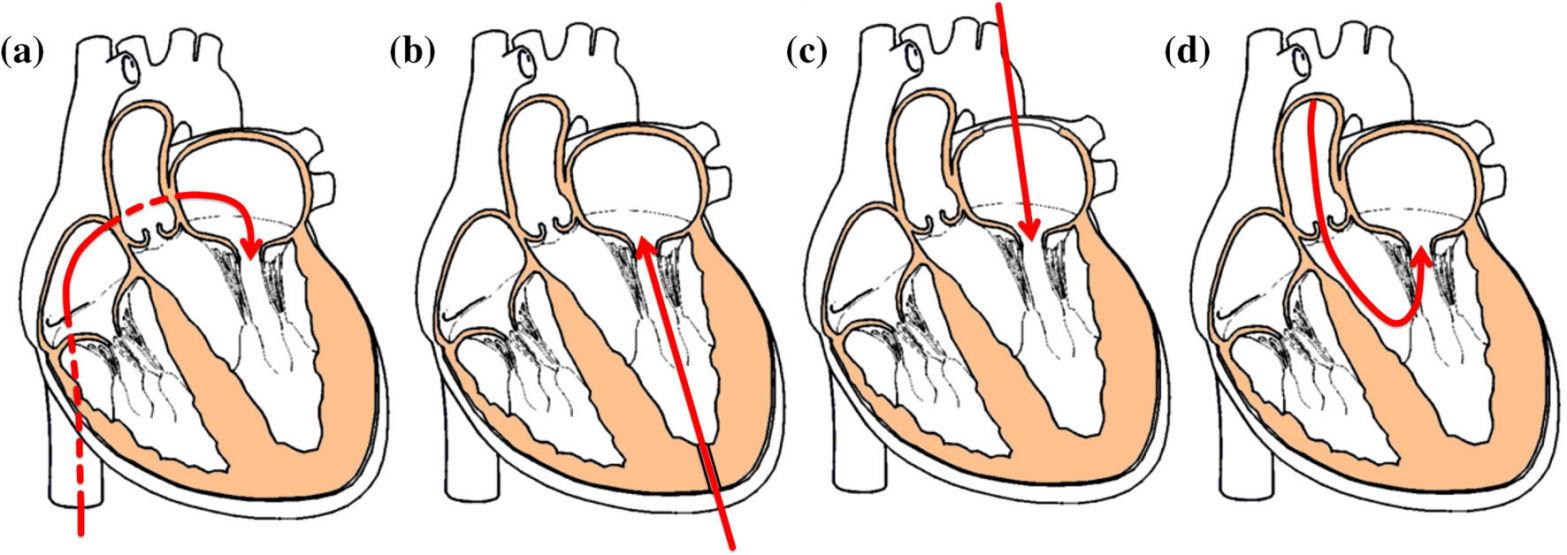
Percutaneous mitral valve delivery. (a) Transseptal, (b) transapical, (c) left atriotomy, and (d) Transaortic

With sufficiently low valve profiles, endovascular retrograde approach allows to conveniently reaching the aortic valve from the femoral vein, after puncturing of the inter-atrial septum (Fig. 2a). This approach has been very popular in the early experience with TAVI devices, because the larger dimensions and greater elasticity of the veins allows the passage of relatively large collapsed stents. In the case of the mitral valve, this is the most favorable access, because it does not require navigation through the subvalvular apparatus. However, this procedure requires the device to fit inside a catheter equal or smaller than 24 F (8 mm diameter), while current valves still require relatively larger catheters of 30–33 Fr (10–11 mm diameter), with the exception of the *MitrAssist*, for which a delivery system diameter of 18 Fr (6 mm diameter) is declared. Currently, only three of the devices have achieved a percutaneous delivery; *CardiAQ Prosthesis, NaviGate Cardiac Structures* and *MiVAR*, all but the later use a transseptal approach.^16^

The transapical route (Fig. 2b), widely used for the implantation of aortic valves, allows the passage of larger valves; and therefore is currently the most adopted approach. However, access to the mitral valve requires navigating the delivery system through the subvalvular apparatus. To prevent the device becoming entangled in the chordae tendineae, expert interpretation of the fluoroscopic and transesophageal echocardiographic guidance (TEE) is required. Despite this challenge, the transapical approach has been used for implanting the *CardiAQ Prosthesis, FORTIS* and *Tiara* 4 and is necessary for the anchoring systems of the *Tendyne, MitralSeal* and *Endovalve*.

A left atriotomy (Fig. 2c) is required to implant the *Double*-*Crowned Mitral Valve Implantation,* *Gorman, HighLife* and *Medtronic* valves. This approach involves an incision of approximately 10 cm, making it the most invasive access for transcatheter valves implantation.

A unique choice of surgical approach is adopted for the *MitrAssist*, which currently uses a transaortic implant (Fig. 2d) where a minimally invasive surgical incision into the aorta is made to insert the device.

#### *In vivo* Evaluation

Evaluation of transcatheter prosthetic mitral valves in large animals has been reported for the *CardiAQ Prosthesis*, *Cardiovalve*, *Double*-*Crowned Mitral Valve Implantation*, *Endovalve*, *FORTIS,* *Gorman*, *HighLife*, *Medtronic*, *MitrAssist*, *Tendyne* and *Tiara*, similarly indicating that the approach is feasible and promising, whilst highlighting their current deficiencies.^3^,^4^,^16^,^23^,^27^,^37^,^55^ A common failure mode of the devices was inadequate anchoring mechanisms, resulting in device migration, which was also one of the main causes of LVOTO. However some of these failure modes may be due to inappropriate sizing. For example, the relatively high rate of PVL in the chronic in animal evaluation of the *Tiara* was attributed to only one size device being available for the range of native valves.^16^ The *MitralSeal* is routinely employed for veterinary applications in dogs, which are characterized by similar mitral valve pathologies to humans.^44^

Generally, in animal experience with TMVIs indicate that the current devices require substantial design improvements, namely to position and anchor the devices. These improvements may partly be achieved by developing appropriate algorithms for selecting the size of the device to be implanted for a particular native valve. For the *Tiara* device, implants in human cadavers are reported,^4^ which demonstrated appropriate geometric positioning.

In human evaluation of minimally invasive mitral valve replacements has mainly occurred in the last couple of years. In 2012, the *CardiAQ* prosthesis was the first to be implanted in a human, followed by the *Tendyne* in 2013, and the *Tiara* and *FORTIS* in 2014. The *Cardiovalve, Double*-*Crowned Mitral Valve Implantation*, *Endovalve*, *Gorman, HighLife, Medtronic, MitrAssist* and *MiVAR* all remain in animal trials, whilst no *in vivo* data are reported for the *NaviGate Cardiac Structures* yet.

The first-in-man experience of the *CardiAQ Prosthesis* was performed by the team of Lars Søndergaard at the Heart Centre in Copenhagen, Denmark, in June 2012. The valve was implanted into an 86-year-old high-risk patient suffering from severe MR. The device successfully alleviated the patient’s severe regurgitation, although some remained and 3 days later the patient died from multi-organ failure.^49^

In the following year two *Tendyne* valves were implanted at the French Hospital in Asuncion, Paraguay.^16^ They were successful in reducing the MR grade from IV to a grade I in one patient and completely eliminated it in the other.

The *Tiara* was implanted into two patients at St. Paul’s Hospital, Vancouver, British Columbia, Canada^16^ at the beginning of 2014 and successfully eliminated MR as well as achieving improved LV stroke volumes.

Later in 2014, a total of five *FORTIS* valves were implanted, four by the Heart Team at St. Thomas’ Hospital in London, UK and one at St Michael’s Hospital in Toronto, Canada.^5^ The patients had presented with severe mitral valve disease and showed promising initial recovery after the implantation of *FORTIS*, although 3 of the patients died between 4 and 76 days after the procedure. Failure modes and causes of death have not been reported.

The human trials have demonstrated the potential of TMVIs to diminish MR to a similar level achieved by surgical replacements, however the long term follow up of these implantations is still on going.

## Regulatory Issues

Prior to reaching the market, the safety and efficacy of any prospective heart valve substitute must be ascertained by means of *in vitro* and *in vivo* investigations. Guidelines and recommendations for the qualification of the design and manufacture of permanent prosthetic valve devices are provided by the International Standards ISO 5840—*Cardiovascular implants*—*Cardiac valve prostheses*. This imposes the design and minimum performance specifications for aortic and mitral valves, and outlines the approach required to assess the properties of the prostheses and their materials, and those for the *in vivo* evaluation. The standard, first enforced in 1984 and initially based on a ‘requirement based’ approach, has recently adopted a ‘risk based’ approach (ISO 5840:2005), more in line with FDA guidance and better suited for the recent transformative developments of the technologies. The substantial technological and functional differences between surgical and minimally invasive valve prostheses have driven the drafting and publication of an additional updated part of the standard, ISO 5840-3:2013, specifically created for the qualification of transcatheter heart valve substitute and valid since March 2013.

Most of the specifications and assessment approaches described in the International Standards ISO 5840:2005 and ISO 5840-3:2013 are applicable to minimally invasive mitral valve replacements. However, as they are based on the existing clinical evidence, mainly available only for surgical valves and TAVI, revision may be needed to better address this emerging class of devices.

### Diastolic Performance

The current standards prefer the measurement of the effective orifice area (EOA) to assess the hydrodynamic performance of valves, during the forward flow phase (diastolic phase for the mitral valve). However, due to its larger orifice, the EOA is a less critical parameter for the healthy function of the mitral valve compared to the aortic valve, and is often sacrificed in favor of better leaflet coaptation. For example, in the edge-to-edge repair technique, the EOA of the mitral valve can be reduced by as much as 60%, without detrimental consequences. Therefore, assessment based on the effective surface area using the proximal isovelocity surface area (PISA) method^34^,^46^ may be more relevant for the intraoperative (and *in vitro*) evaluation of mitral devices.

### Systolic Performance

The performance of the valve during the closing and closed phase is verified and controlled by limiting the regurgitant fraction. The ISO 5840-3:2013 acknowledges the inevitability of higher degrees of paravalvular leakage in transcatheter devices, accepting larger regurgitant fractions (up to 25% at a CO of 5 L/min, for large sizes) than for corresponding surgical valves. This is compensated by more severe demands in terms of EOA.

As discussed above, though this approach is acceptable for TAVIs, in the case of the mitral valve, increases in EOA would possibly be unnecessary and insufficient to compensate significant levels of leakage, which should instead be mitigated.

### Risk Management Aspects

The potential hazards, associated failure modes and subsequent evaluation methods currently described in the ISO 5840-3:2013 (Annex G) are defined based on the TAVI experience. In order to better reflect the lesson learnt from current *in vivo* experiences with TMVI, the risks associated with unintended anatomical interactions should also consider failure modes such as: *left ventricular out flow tract obstruction (LVOTO)*, which can lead to heart failure and death; *left circumflex (LCx) artery compression*, which would inhibit the blood supply to the posterolateral left ventricle and anterolateral papillary muscle; and *coronary sinus (CS) compression,* which would hinder the deoxygenated blood flow from the heart muscles to the right atrium.

### Animal Evaluation

The use of animal models to evaluate TAVI devices has severe limitations. As stated in the ISO 5840-3:2013, the soft nature of the tissue present in the animals’ aortic valve is not representative of the stiffer calcified aortic valves that would be present in typical human patients. As a result, preclinical *in vivo* tests cannot assess the migration of TAVI solutions. However, the softer anatomical site is arguably more representative of a regurgitant mitral valve, and therefore the function of animal evaluation for TMVIs should include assessment of migration of the devices. This will require a more strict identification of representative animal sizes, in order to ensure that the implants provide the most significant information.

## Discussion and Conclusion

After the rapid development of pulmonary and aortic transcatheter heart valve implantations, the advantages of percutaneous procedures are finally being transferred to the functional replacement of the mitral valve. The experience gained with TAVI devices is resulting fundamental in the development of this approach, which though presents unique challenges requiring new technical solutions, due to the more complex morphology and function of the mitral valve.

Similarly to the first generation of percutaneous aortic valve, PVL and embolization remain major hurdles, whose impact is amplified by the more severe transvalvular pressures and valve dynamics. The accuracy of medical imaging technologies and sizing criteria will play a major role in reducing these complications, by allowing the selection of the most suitable TMVI for each patient. In fact, for a correct fitting, most of the valves under development require proper geometrical matching with the irregular mitral annulus (normally characterized by an asymmetric bean-shape profile, laying on a saddle-shape surface), as well as the subvalvular structures and the heart chambers. All these components are characterized by significant variations of their dimensions during the cardiac cycle, due to the dynamic nature of the mitral valve apparatus and surrounding anatomy. Therefore, for each prosthetic valve, it is essential to identify what parameters are the most relevant for ensuring proper anchoring and sealing. Accurate and comprehensive sizing algorithms will need to be developed, to ensure the safety and proper functionality of TMVIs. This will necessarily require continuous revision and refining, based on the *in vivo* experience.

Future designs will also need to focus on reducing the collapsed profile of the device, enabling a truly endovascular access. This will foreseeably follow the developmental trend that has characterized aortic solutions, which have achieved substantial advances in reducing the size of the loaded valve and delivery system, offering now alternative implantation approaches with reduced vascular injury. An important feature to inherit from the latest generation of TAVI will be the ability to reposition the valve during the implantation procedure. In fact, as most of the anchoring approaches adopted for the mitral application require a precise axial and angular positioning, inappropriate release is more likely to lead to functional complications and increased PVL.

Concerns about safety and durability in the functionally more stressed mitral position will be relieved only after medium and long term clinical outcome, but the positive results of TAVI devices suggest that metal frames can be suitable to withstand heavy loading conditions. Also, as for TAVI, transcatheter mitral valves might result into a high rate of silent cerebral ischemic lesions. These, in fact, appear to be independent from catheter manipulation or the severity of calcification, and possibly associated with the hemodynamic disturbance produced by the valve-in-valve configuration.^19^

In order to be established as a sustainable therapy, it is essential that transcatheter mitral valve replacement fully demonstrates its safety and efficacy, guaranteeing at the same time affordable costs. In fact, contrary to TAVI devices, which only compete with other replacement strategies, mitral valves will have to operate in a more aggressive commercial climate, contending the role to a range of surgical and transcatheter, replacement and repair solutions, with various degrees of invasivity. However it can be envisaged that, similarly to what has happened for aortic valve therapies, also currently established mitral treatments will strongly benefit from the novel technical solutions developed for TMVI. In fact, the dimensional reduction of the supportive structure of the leaflets and the availability of collapsible sutureless mitral valves may contribute to the development of safer and less invasive surgical applications.

In conclusion, first experiences with transcatheter mitral valve replacement have been very encouraging, demonstrating the feasibility and the potential benefit of the approach. Undoubtedly, substantial progresses will be needed in order to overcome current limitations and establish this solution as a competitive treatment. The process will have to be assisted by regulatory standards that better reflect the specific clinical needs and the recent experiences with this approach.
